# Supplementary material for: Enhanced JunD/RSK3 signalling due to loss of BRD4/FOXD3/miR-548d-3p axis determines BET inhibition resistance
Source: Nat Commun. 2020 Jan 14;11:258. doi: 10.1038/s41467-019-14083-4 (PMC6959298; doi:10.1038/s41467-019-14083-4)

**Supplementary Information**

# Enhanced JunD/RSK3 signaling due to loss of BRD4/FOXD3/miR-548d-3p axis determines BET inhibition resistance

**Tai *et al*.**

# Supplementary Figure 1

**A**

| Gene Name | Log2 Fold Change | *P*-value |
|---|---|---|
| RPS6KA2 | 2.731328499 | 0.00232 |
| PLK3 | 2.418385403 | 0.006036 |
| PIP5K1B | 3.970929411 | 0.010216 |
| ILK | 3.141891774 | 0.012141 |
| ADCK1 | 2.207899783 | 0.014965 |
| LTK | 2.042264218 | 0.020246 |
| CKB | 1.980956344 | 0.021735 |
| SGK2 | 6.259420539 | 0.022775 |
| ADCK5 | 1.955142116 | 0.024327 |
| MAP2K3 | 1.927121536 | 0.025293 |
| AATK | 2.128107774 | 0.026805 |
| DGKQ | 1.910891303 | 0.026978 |
| ITPKC | 1.940212915 | 0.027268 |
| NIM1K | 2.155377828 | 0.028192 |
| SBK1 | 2.052260362 | 0.031294 |
| MKNK2 | 1.832889131 | 0.032934 |
| HYKK | 2.485589481 | 0.034088 |
| PIM2 | 1.718013558 | 0.046533 |

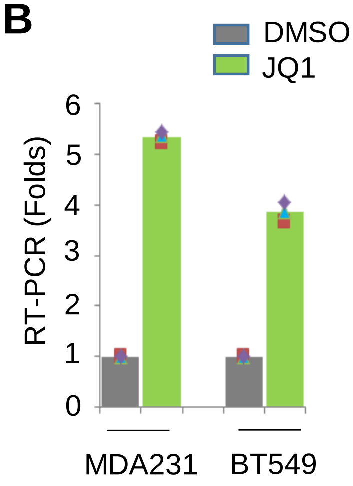


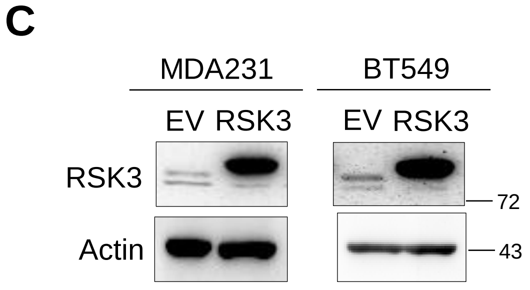


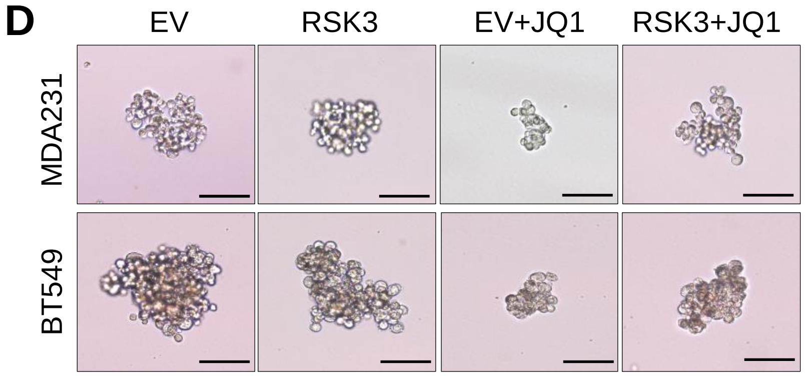


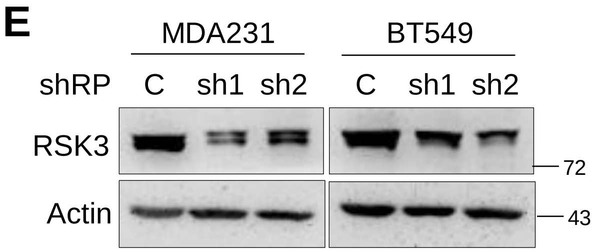


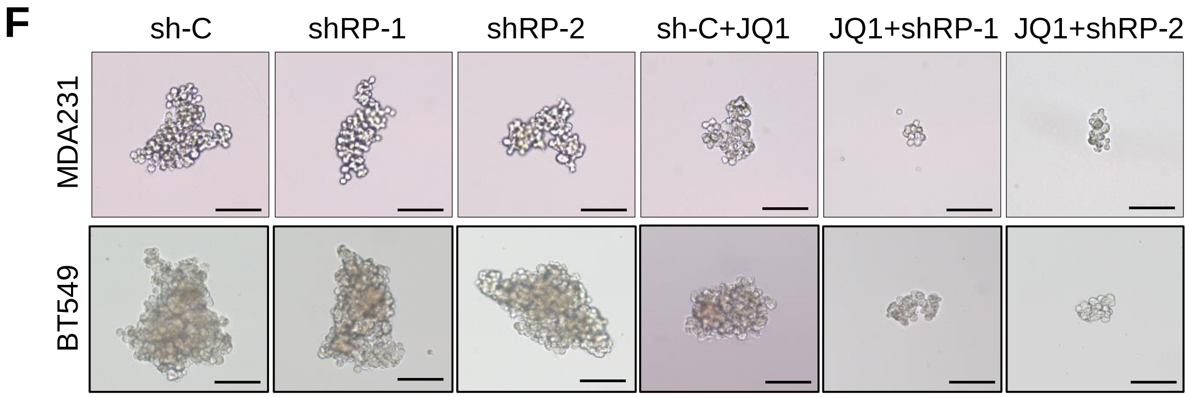


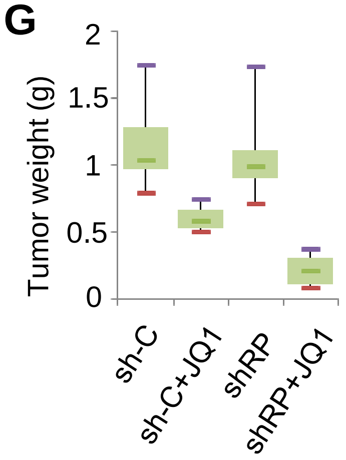

**Supplementary Figure 1. Elevated RSK3 expression is responsible for BETi resistance**

**(A)** List of kinase-encoding genes derived from RNA-sequencing analysis that up-regulated in JQ1 treated MDA-MB-231 cells compared with control cells.

**(B)** *RPS6KA2* mRNA levels were determined by real-time PCR in the presence of JQ1 treatment for 24 h.

**(C**) The levels of RSK3 were detected in the vector control and RSK3-overexpressing clones.

**(D)** Tumoursphere formation in RSK3-overexpressing BLBC cells and their vector controls was detected with or without JQ1 (1 μM) treatment. Typical pictures of tumoursphere are shown. Scale bar is 100 μM.

**(E**) The levels of RSK3 protein were detected in the vector control and shRNA-mediated *RPS6KA2*-knockdown BLBC clones by western blotting.

**(F)** Tumoursphere was counted in *RPS6KA2*-knockdown BLBC cells and their vector controls in the absence or presence of JQ1 (1 μM). Typical pictures of tumoursphere are shown. Scale bar is 100 μM.

**(G)** Measurement of tumour weight derived from vector or *RPS6KA2* -knockdown MDA-MB-231 which treated with vehicle or JQ1.

# Supplementary Figure 2

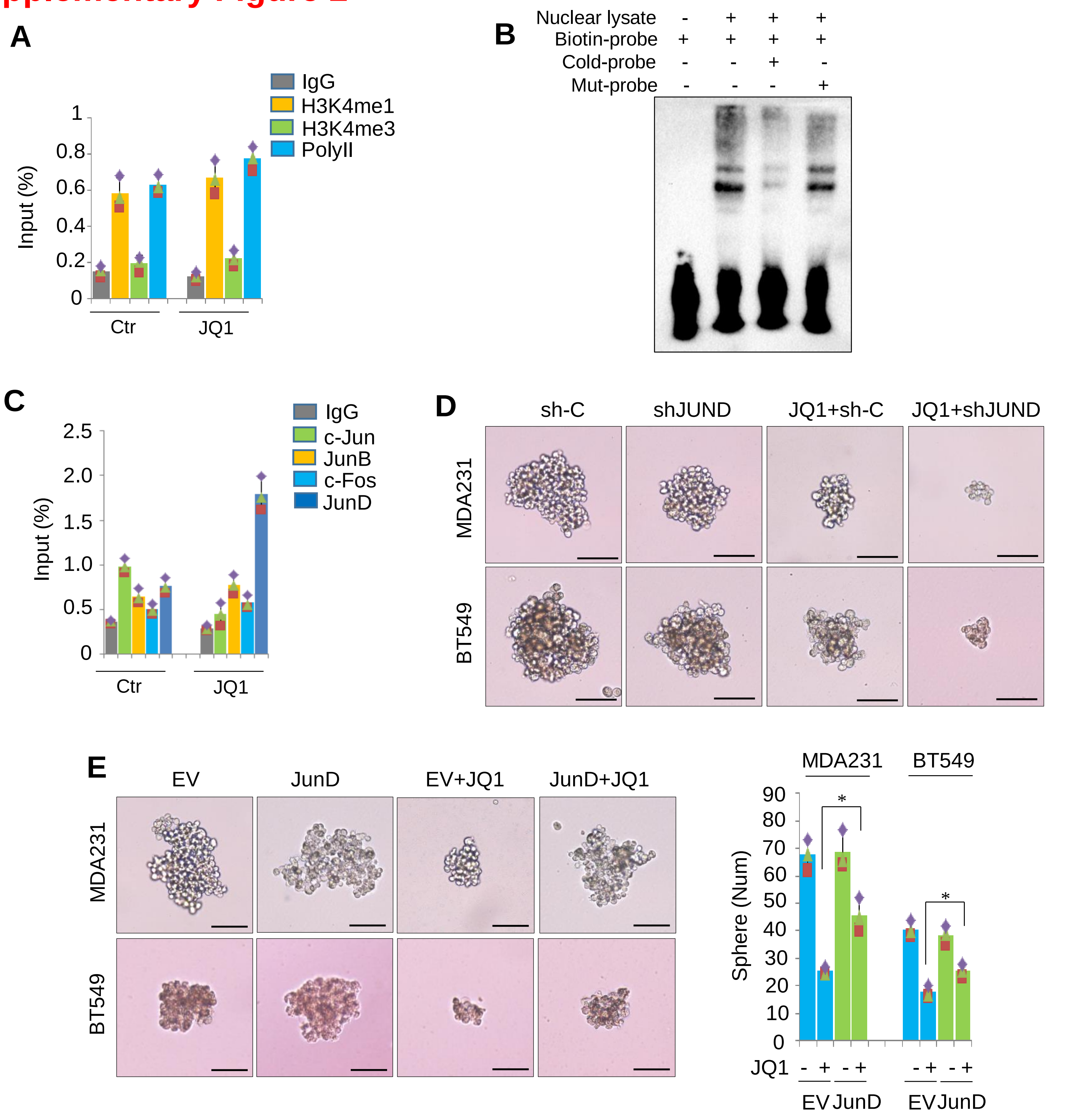


**Supplementary Figure 2. JunD-dependent *RPS6KA2* transcription mediates BETi resistance**

**(A)** ChIP assay performed by use of antibodies of H3K4me1, H3K4me3 and RNA polymerase II.

**(B)** EMSA assay was done using LightShift™ Chemiluminescent EMSA Kit (Thermo Fisher, #20148), the biotin-labelled DNA probes are 5'-GTCTTTTATGAGTGACTCTCCTAGCTTTTT-3' and 5'-AAAAAGCTAGGAGAGTCACTCATAAAAGAC-3'. MDA-MB-231 cells were treated with JQ1 (1 μM) for 6 h.

**(C)** ChIP assay performed with antibodies of c-Jun, JunB, JunD and c-Fos.

**(D)** Tumoursphere formation assay was conducted in *JUND*-knockdown MDA-MB-231 and BT549 cells as well as their vector controls. The cells were treated with DMSO or JQ1 (1 μM). Typical pictures of tumoursphere are shown. Scale bar is 100 μM.

**(E)** Measured tumoursphere formation in JunD-overexpressing BLBC cells and their vector controls. The cells were treated with DMSO or JQ1 (1 μM). Typical pictures of tumoursphere are shown. Scale bar is 100 μM. Statistical data (mean ± SD) are shown (n=3, *$P$<0.05, one-way ANOVA).

# Supplementary Figure 3

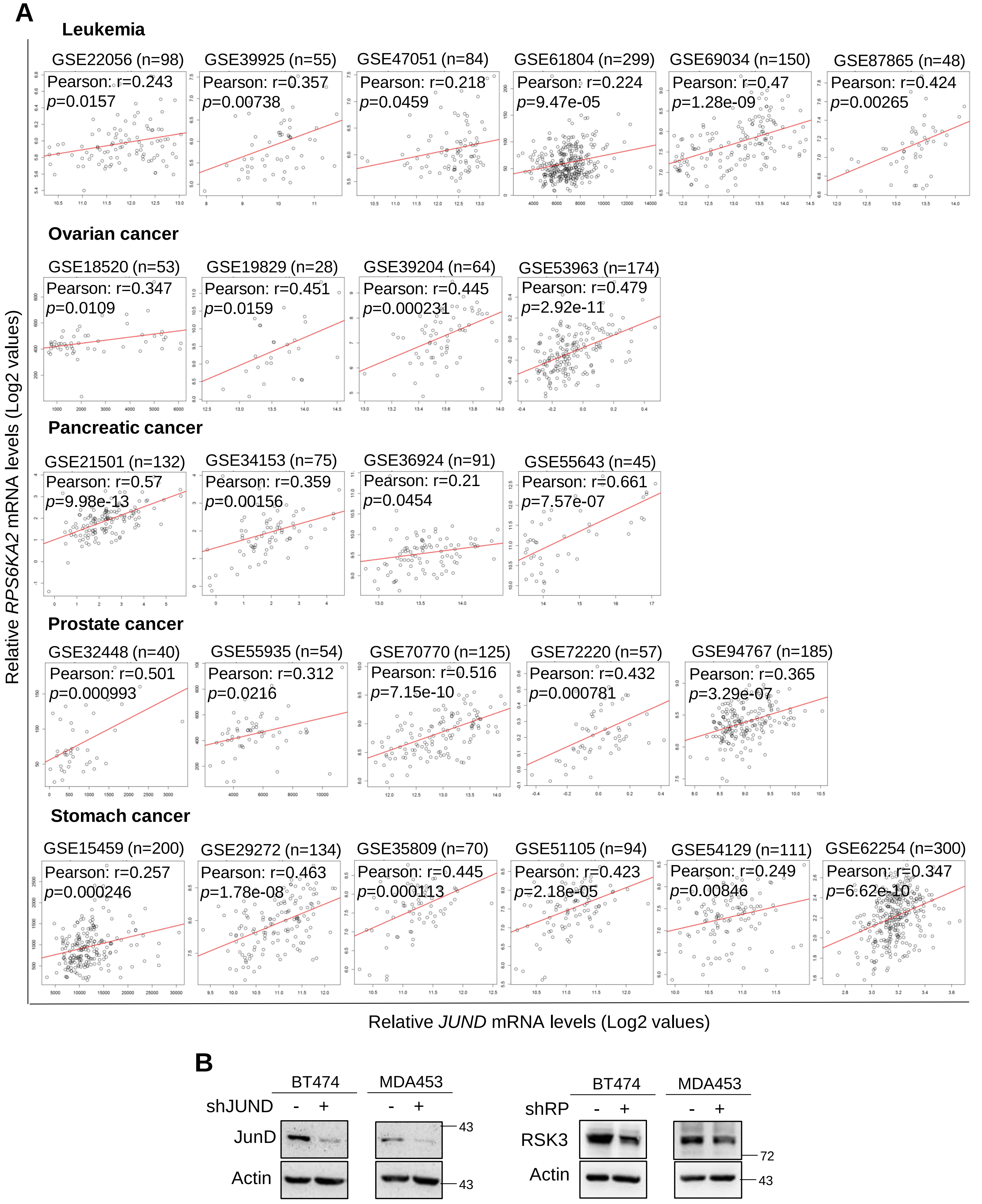

**Supplementary Figure 3. JunD/RSK3 signaling correlates to BET inhibition sensitivity**

**(A)** Correlation analyses of *JUND* and *RPS6KA2* in gene expression datasets from multiple types of cancer. *Pearson* Coefficients of correlation are shown.

**(B)** *JUND* and *RPS6KA2* were knocked down in BT474 and MDA-MB-453 cells.

# Supplementary Figure 4

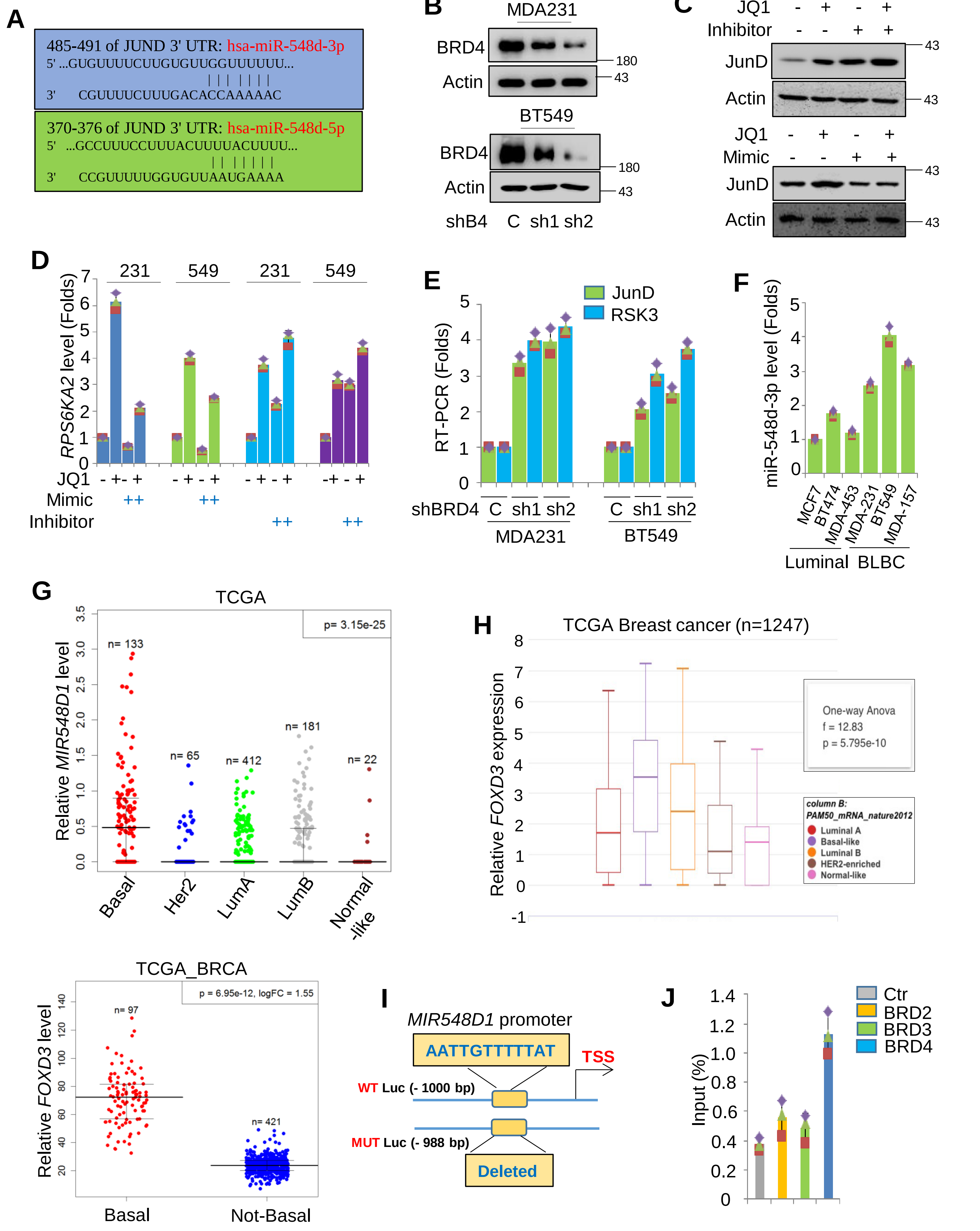

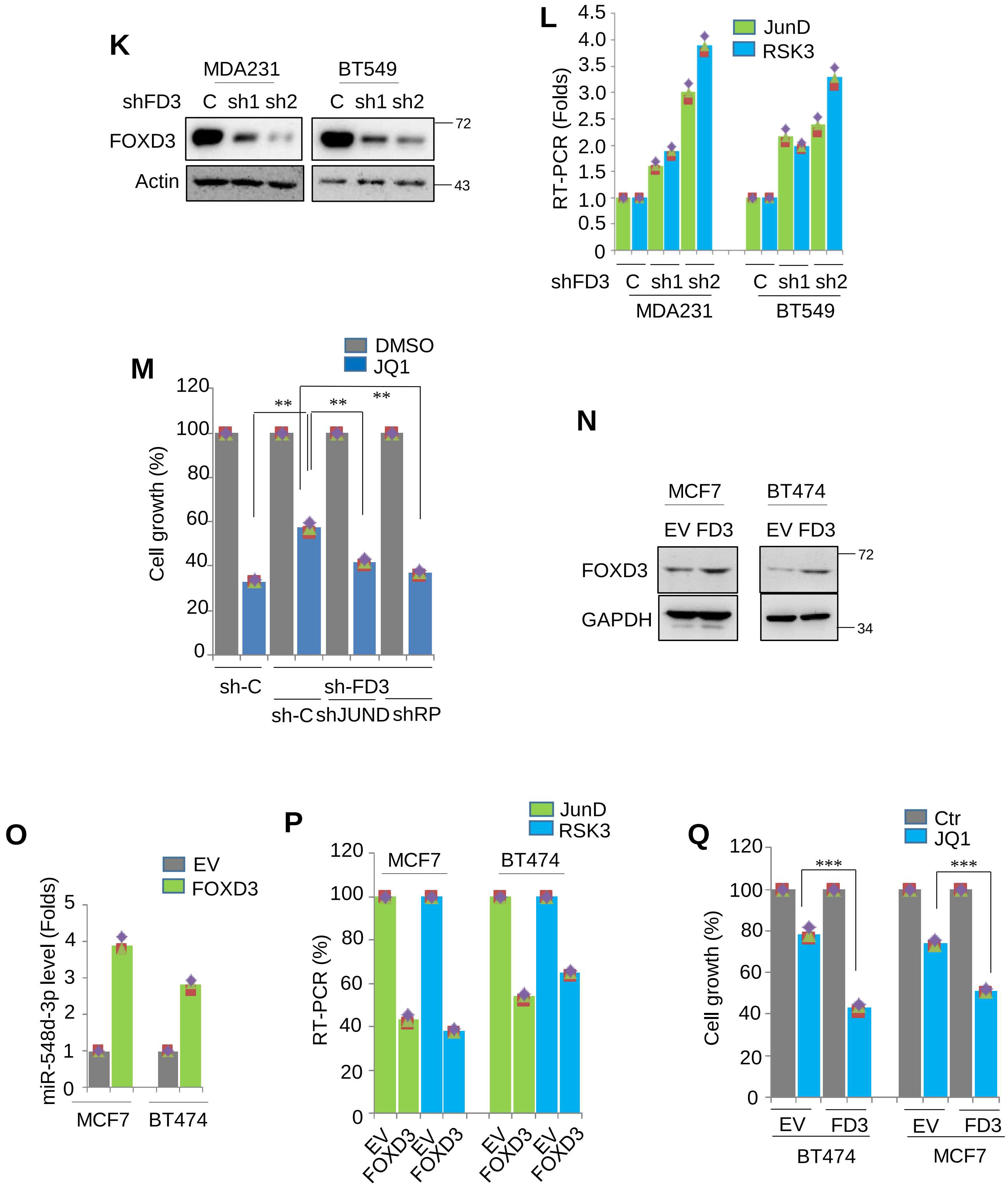

K
MDA231
BT549
shFD3
C sh1 sh2
C sh1 sh2
FOXD3
Actin
72
43
L
RT-PCR (Folds)
4.5
4.0
3.5
3.0
2.5
2.0
1.5
1.0
0.5
0
JunD
RSK3
shFD3
C sh1 sh2
C sh1 sh2
MDA231
BT549
M
DMSO
JQ1
Cell growth (%)
120
100
80
60
40
20
0
**
**
**
sh-C
sh-FD3
sh-C shJUND shRP
N
MCF7
BT474
EV FD3
EV FD3
FOXD3
GAPDH
72
34
O
miR-548d-3p level (Folds)
5
4
3
2
1
0
EV
FOXD3
MCF7
BT474
P
JunD
RSK3
RT-PCR (%)
120
100
80
60
40
20
0
MCF7
BT474
EV
FOXD3
EV
FOXD3
EV
FOXD3
EV
FOXD3
Q
Ctr
JQ1
Cell growth (%)
120
100
80
60
40
20
0
***
***
EV
FD3
EV
FD3
BT474
MCF7

**Supplementary Figure 4. JQ1 represses BRD4/FOXD3-maintained miR-548d-3p expression**
**(A)** Photograph depicted the recognition sequence of miR-548d-3p and miR-548d-5p on the *JUND* 3'UTR region.
**(B**) Detection of *BRD4* silencing status in MDA-MB-231 and BT549 cells.
**(C**) JunD protein expression was measured in MDA-MB-231 cells in the absence or presence of mimic or inhibitor of miR-548d-3p.
**(D**) *RPS6KA2* mRNA expression was examined in BLBC cells by real-time PCR assay in the absence or presence of mimic or inhibitor of miR-548d-3p.
**(E**) *JUND* and *RPS6KA2* mRNA expression was detected in vector control and *BRD4*-knockdown BLBC clones.
**(F**) The expression of miR-548d-3p was detected in a series of breast cancer cell lines.
**(G**) The expression of *MIR548D1* gene was analysed in TCGA.
**(H**) *FOXD3* mRNA expression was analyzed based on TCGA breast cancer database.
**(I)** Photograph depicted the potential FOXD3 binding site in the *MIR548D1* gene promoter region. Wild-type and deleted *MIR548D1* gene promoter luciferase plasmids are shown.
**(J)** ChIP assay to detect the binding status of BRD2, BRD3 and BRD4 on *MIR548D1* gene promoter.
**(K**) Detection of *FOXD3* silencing in MDA-MB-231 and BT549 cells.
**(L**) *JUND* and *RPS6KA2* mRNA expression was detected in vector control and *FOXD3*-knockdown BLBC clones.
**(M**) Detection of cell growth when vector control and *FOXD3*-knockdown cells were co-silenced with shRNA of *JUND* or *RPS6KA2* (n=3, **$P$<0.01, one-way ANOVA).
**(N**) Detection of FOXD3 overexpression status in BT474 and MCF7 cells.
**(O**) The expression of miR-548d-3p was measured in vector control and FOXD3-overexpressing MCF7 and BT474 cells.
**(P**) *JUND* and *RPS6KA2* mRNA expression was detected in vector control and FOXD3-overexpression clones.
**(Q**) Effect of FOXD3-overexpression on JQ1-mediated growth inhibition in BT474 and MCF7 cells (n=3, ***$P$<0.001, one-way ANOVA).

# Supplementary Figure 5

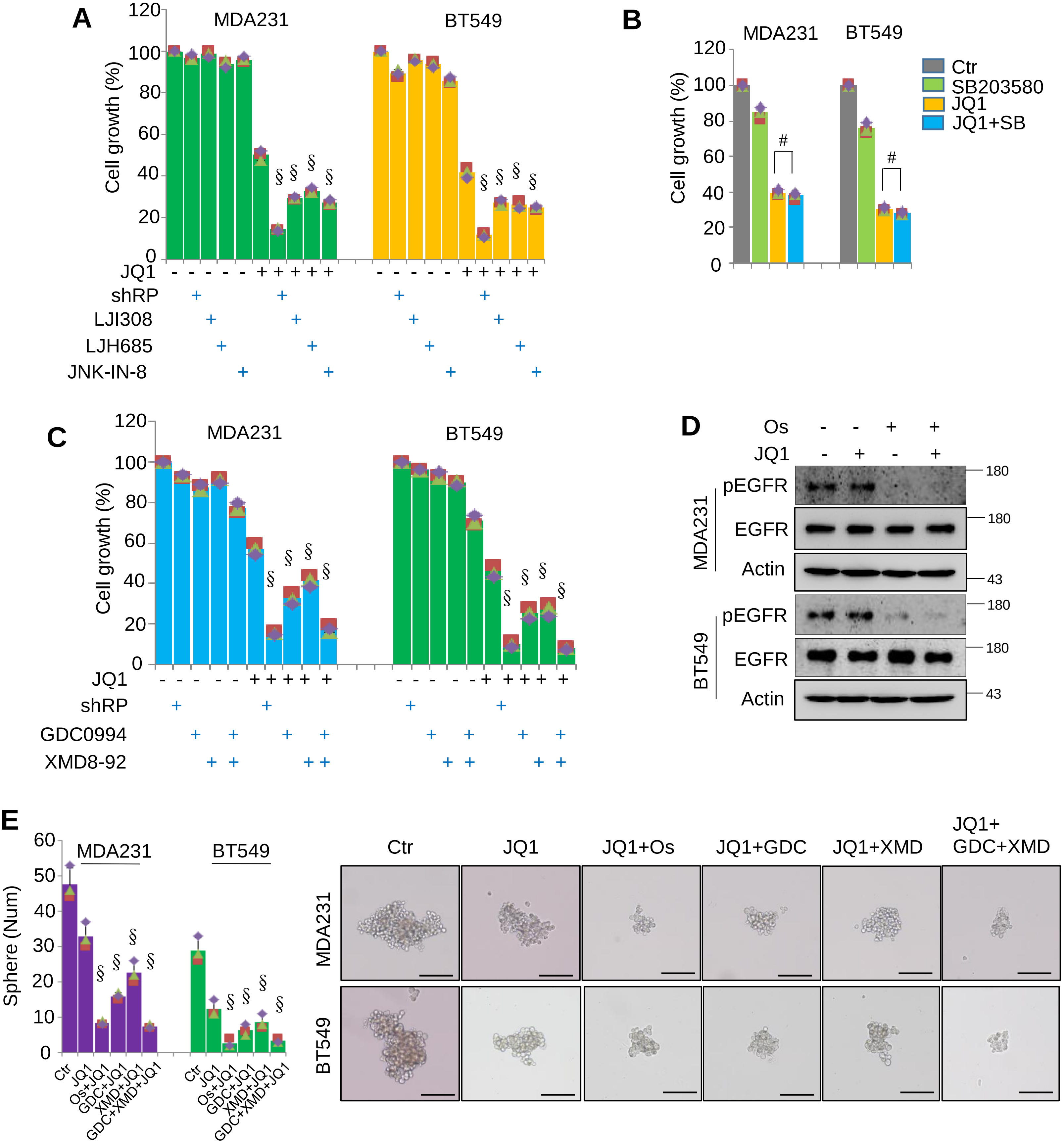

**Supplementary Figure 5. Targeting EGFR/MEK/ERK reverses BET inhibition resistance**

**(A)** Cell growth of MDA-MB231 and BT549 cells was examined by CCK-8 assay in the presence of JQ1 and/or RSKs inhibitors (1 μM) as well as JNK kinase inhibitor JNK-IN-8 (1 μM). *P*-values were calculated when compared between single JQ1-treated samples with those samples treated with JQ1 plus kinase inhibitors. ‘ § ’ indicates significance (n=3, *P*<0.05, one-way ANOVA).

**(B)** Cell growth of MDA-MB231 and BT549 cells was examined by CCK-8 assay in the presence of JQ1 and/or p38 inhibitor SB203580 (1 μM). ‘#’ indicates *P*>0.05 (n=3, one-way ANOVA).

**(C**) Measured cell growth of MDA-MB231 and BT549 cells in the presence of JQ1 and/or GDC-0994 as well as XMD8-92 (1 μM). *P*-values were calculated when compared between single JQ1-treated samples with those samples treated with JQ1 plus kinase inhibitors. ‘ § ’ indicates significance (n=3, *P*<0.05, one-way ANOVA).

**(D**) Phosphorylated and total EGFR levels were detected in JQ1 and/or osimertinib (1 μM) treated BLBC cells.

**(E**) Measured tumoursphere formation in MDA-MB-231 and BT549 cells. Statistical data of numbers of tumoursphere are shown. 1: Ctr; 2: JQ1; 3: JQ1+osimertinib; 4: JQ1+GDC0994; 5: JQ1+XMD8-92; 6: JQ1+GDC0994+XMD8-92 (1 μM). *P*-values were calculated compared between single JQ1-treated samples and samples that treated with JQ1 plus kinase inhibitors. ‘ § ’ indicates significance (n=3, *P*<0.05, one-way ANOVA).

# Supplementary Figure 6

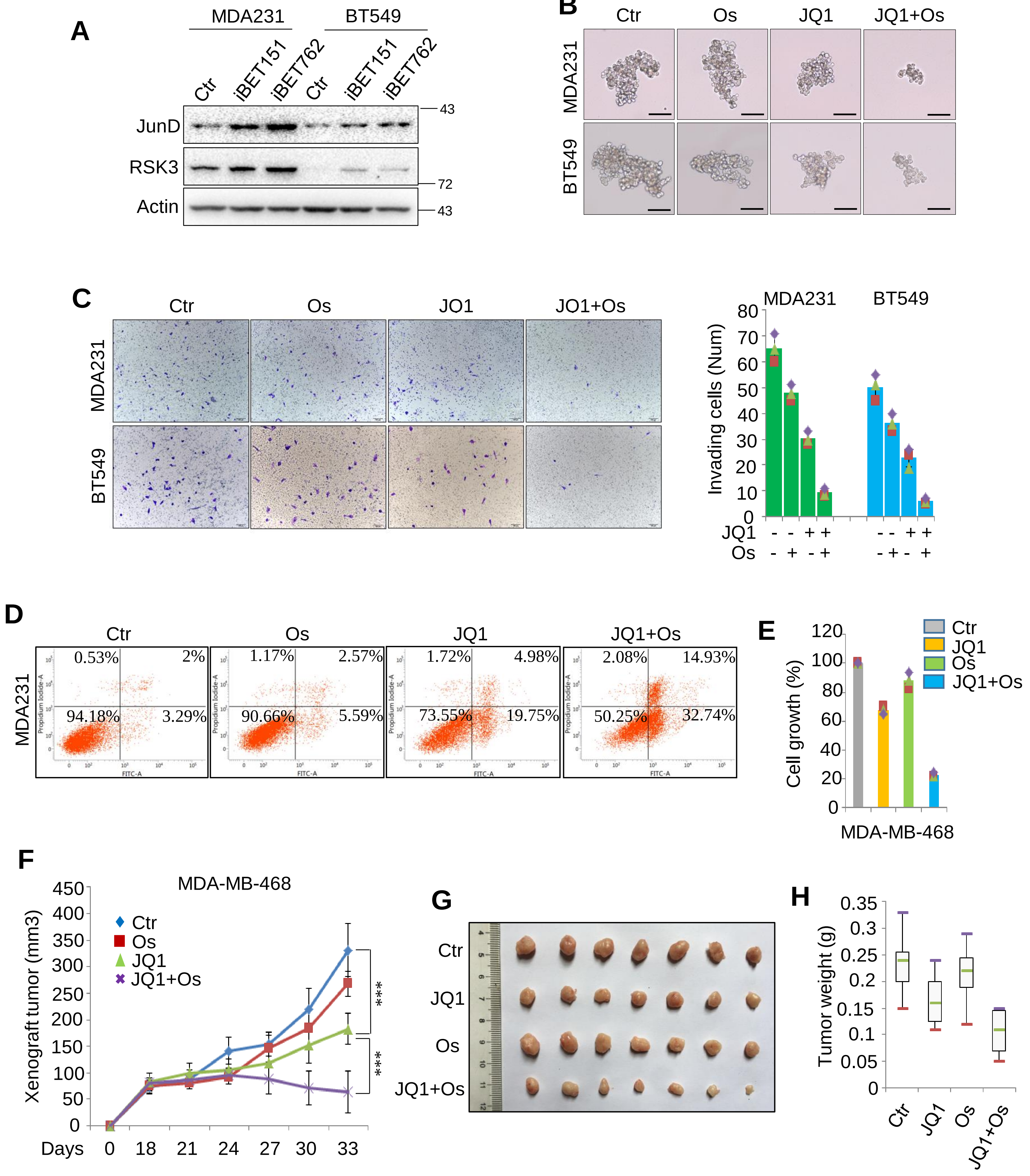

**Supplementary Figure 6. EGFR inhibition overcomes BET inhibition resistance**

**(A**) Western blotting was done to detect the induction of JunD and RSK3 by iBET151 and iBET762 in BLBC cells.

**(B**) Measured tumoursphere formation in MDA-MB-231 and BT549 cells in the presence of JQ1 and/or osimertinib. Typical pictures of tumoursphere are shown. Scale bar is 100 μM.

**(C**) Cell invasive ability of MDA-MB-231 and BT549 cells was tested in the presence of JQ1 and/or osimertinib. Typical pictures of invading cells and statistical data are shown. Statistical data (mean ± SD) are shown. Scale bar is 100 μM.

**(D**) FITC/annexin V FACS analysis was done in JQ1 and/or osimertinib treated MDA-MB-231 cells. A representative experiment is shown.

(**E**) MDA-MB-468 cells were treated with JQ1 (1 μM) and/or osimertinib (1 μM), CCK8 assay was used to detect the effects.

**(F-H**) MDA-MB-468 derived xenograft mice were separated into four groups, which respectively administered with vehicle control, osimertinib (10 mg/kg), JQ1 (35 mg/kg), osimertinib plus JQ1.**F**, Growth curves of xenograft tumour are shown; **G**, Photographs of tumours; **H**, Tumour weight. (n=7, ***$P$< 0.001, one-way ANOVA).

# Supplementary Figure 7

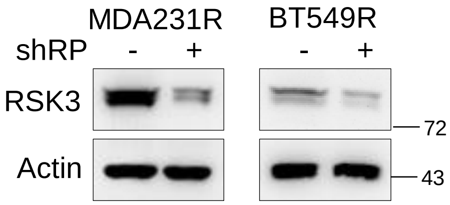


**Supplementary Figure 7. JQ1-resistant BLBC cells are sensitive to combined therapies**
The expression of *RPS6KA2* was knocked down in MDA-MB-231 and BT549 JQ1-resistant clones.

# Supplementary Table 1

| Gene | Assay | Sequence |
|---|---|---|
| JUND | RT-PCR | catcgacatggacacgcag |
| JUND | RT-PCR | agctccgtgttctgactctt |
| RPS6KA2 | RT-PCR | GGGGATCCTGTTGTACACCA |
| RPS6KA2 | RT-PCR | GCGTCAGATATCGAGTCCCA |
| RPS6KA2 | ChIP (specific) | CCTTCTTCCACAACAGAGCAC |
| RPS6KA2 | ChIP (specific) | CCTGAAGGCAATGGGAAAGA |
| RPS6KA2 | ChIP (Non-specific) | AACCAGCATGTGTCAGCAAG |
| RPS6KA2 | ChIP (Non-specific) | GAGCAGCTCAGAAAATGGGG |
| MIR548D1 | ChIP | CACCCGGCCAGTATTAAGTAC |
| MIR548D1 | ChIP | TCCCTGGTGTGAATCTTTAGAAT |

**Full scans of western blots**

**Enhanced JunD/RSK3 signaling due to loss of BRD4/FOXD3/miR-548d-3p axis determines BET inhibition resistance**

**Tai *et al*.**

**Figure.1A**

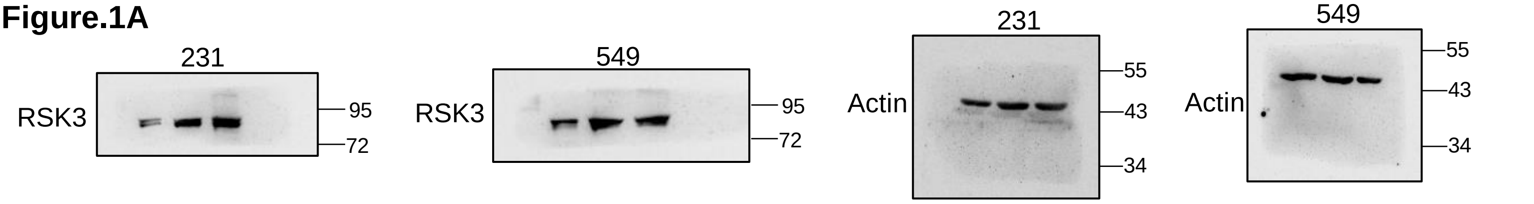


**Figure.2A**

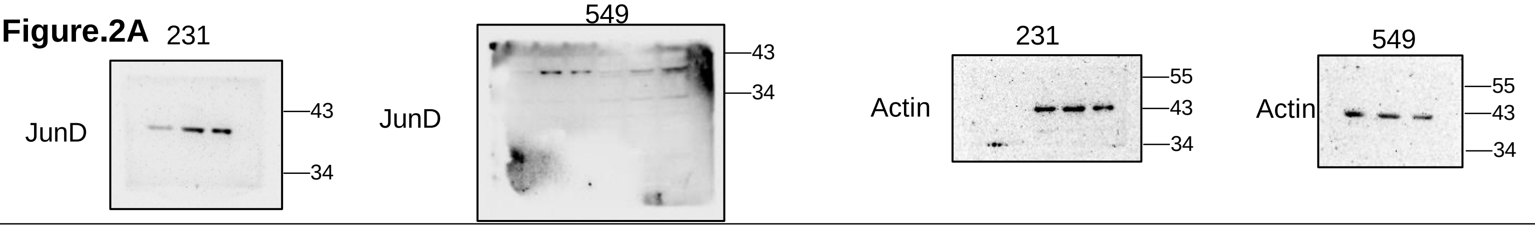


**Figure.2F**

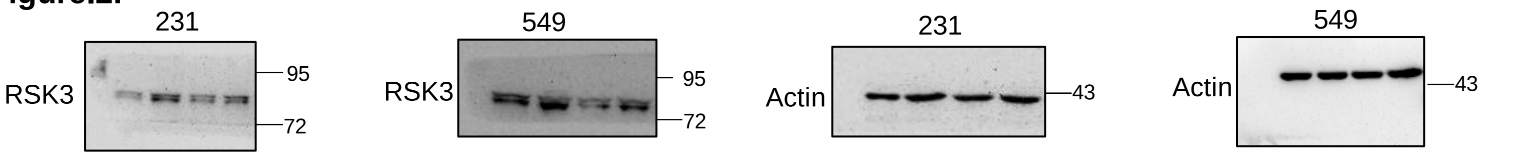


**Figure.2J**

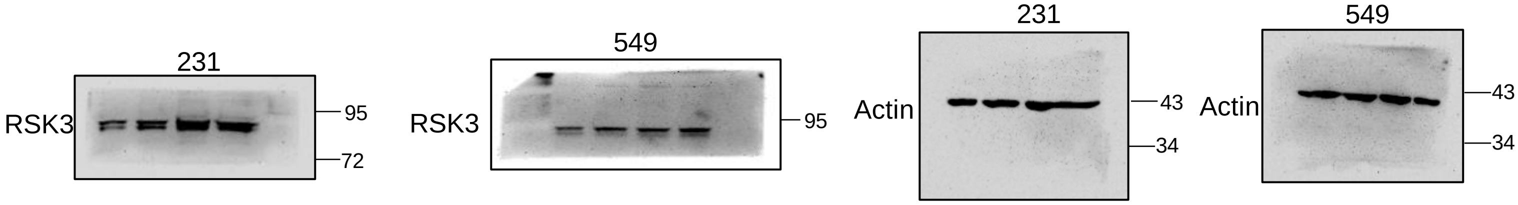


**Figure.3D**

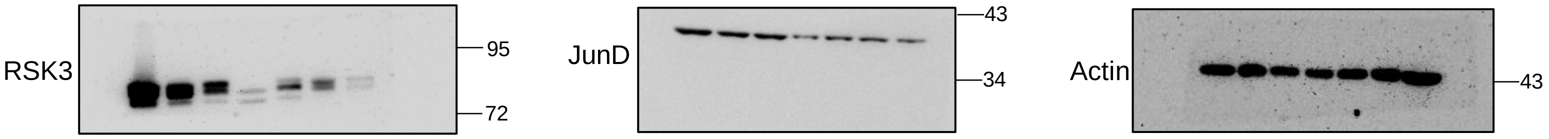


**Figure.4I**

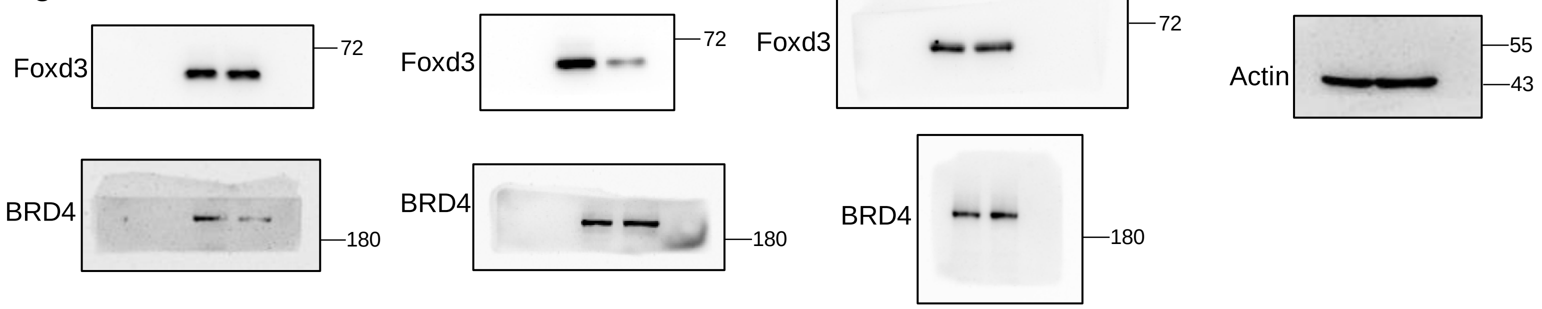

**Figure.5B**

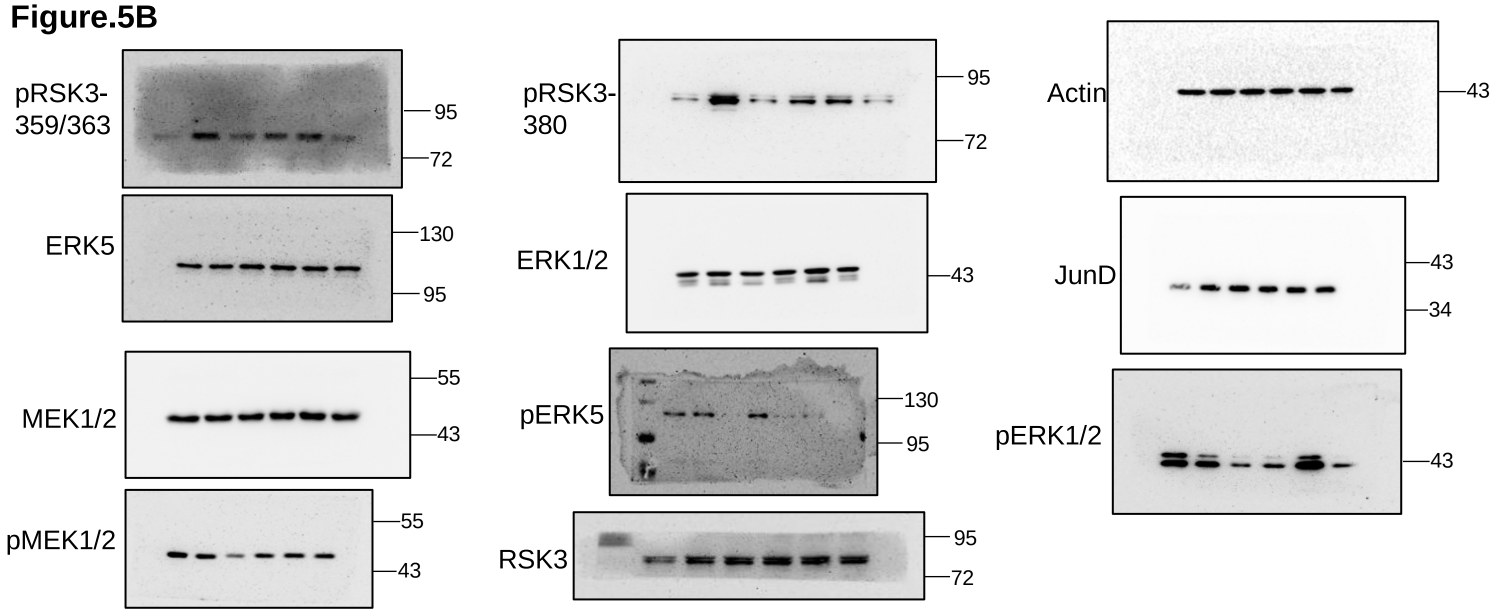


**Figure.6H**

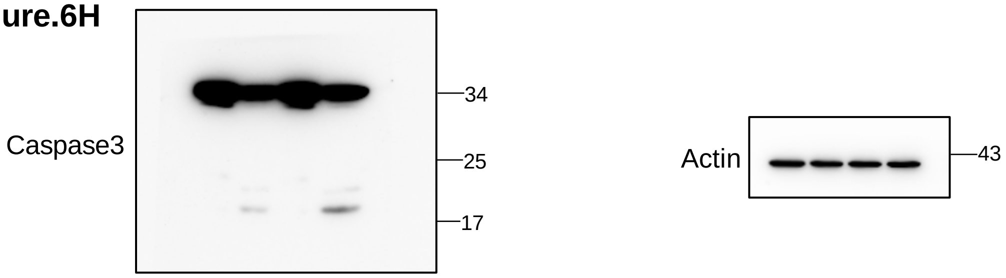


**Figure.7B**

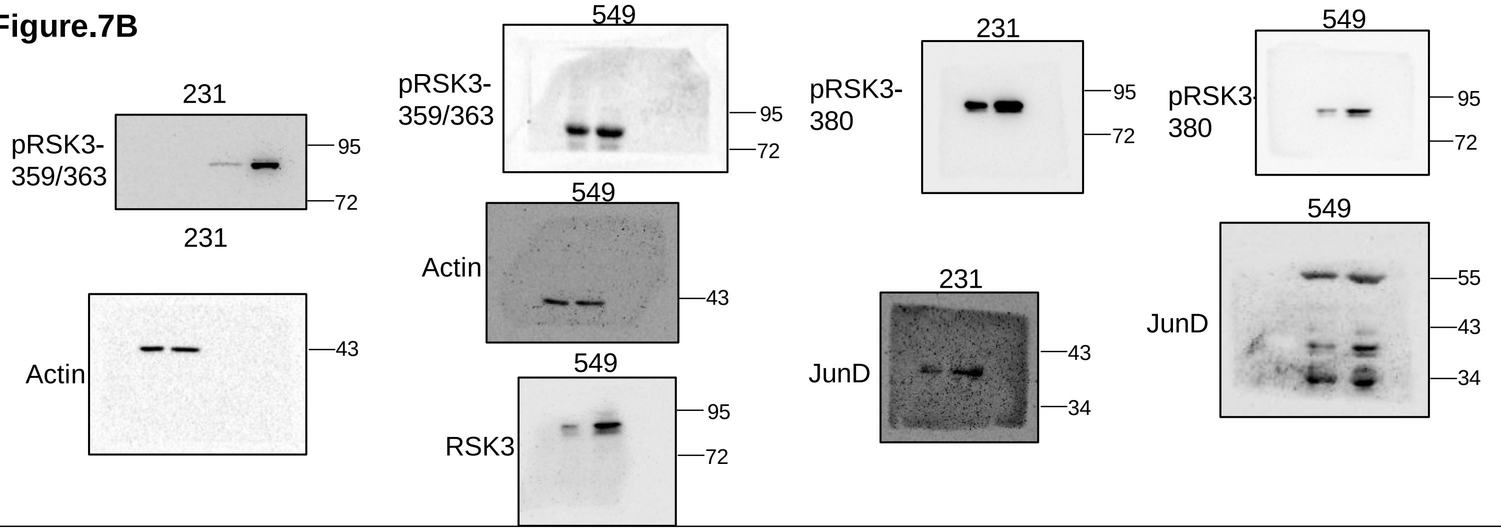


**Supplementary Figure.1C**

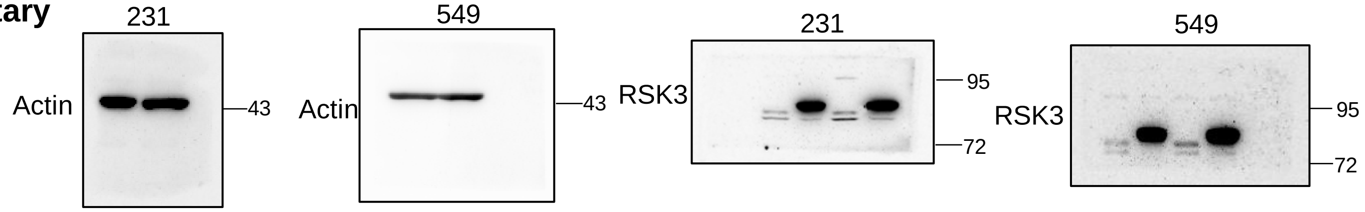


**Supplementary Figure.1E**

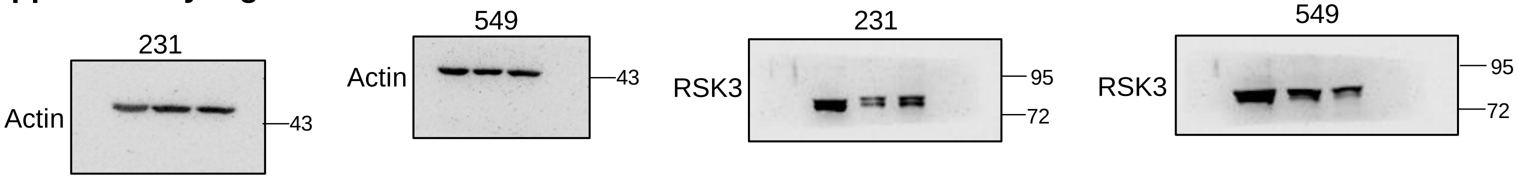

## Supplementary Figure.4B

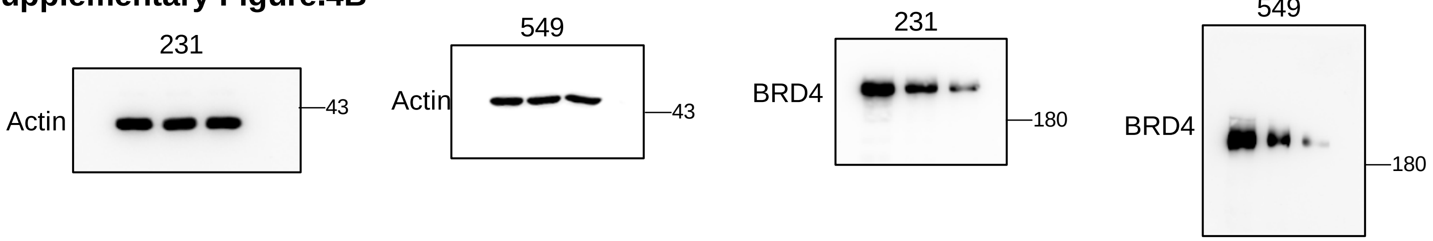


## Supplementary Figure.5D

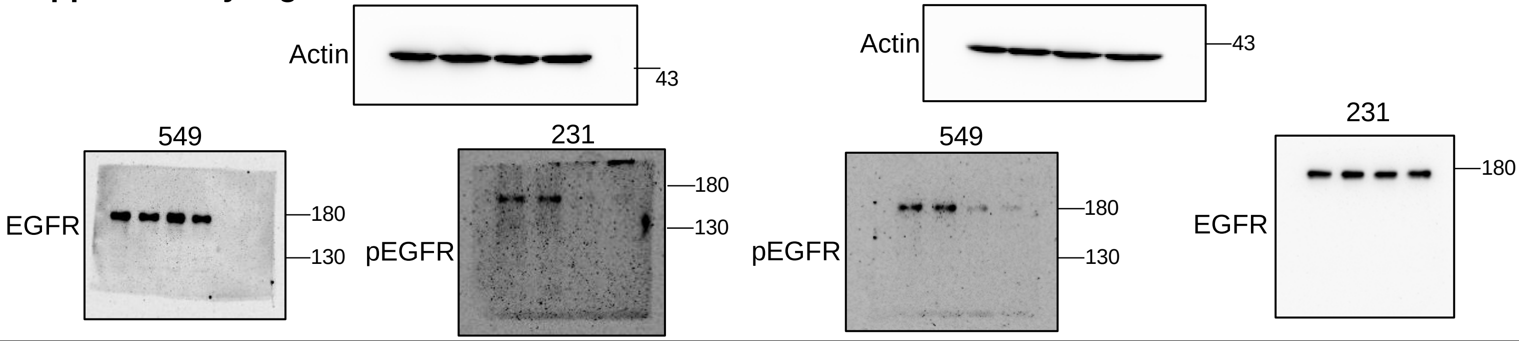


## Supplementary Figure.6A

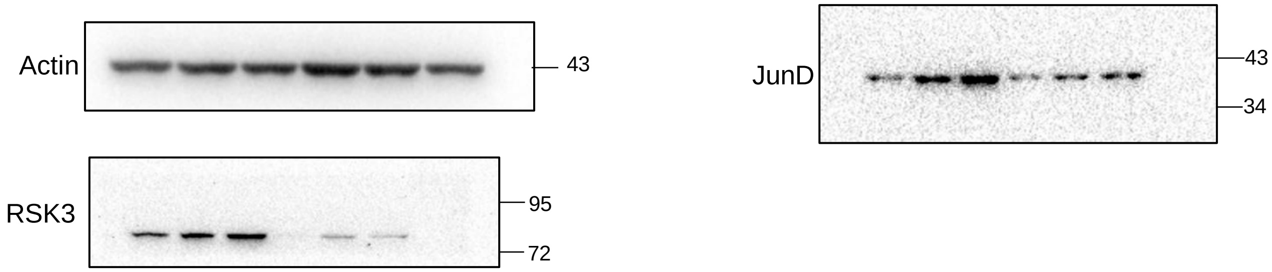


## Supplementary Figure.7

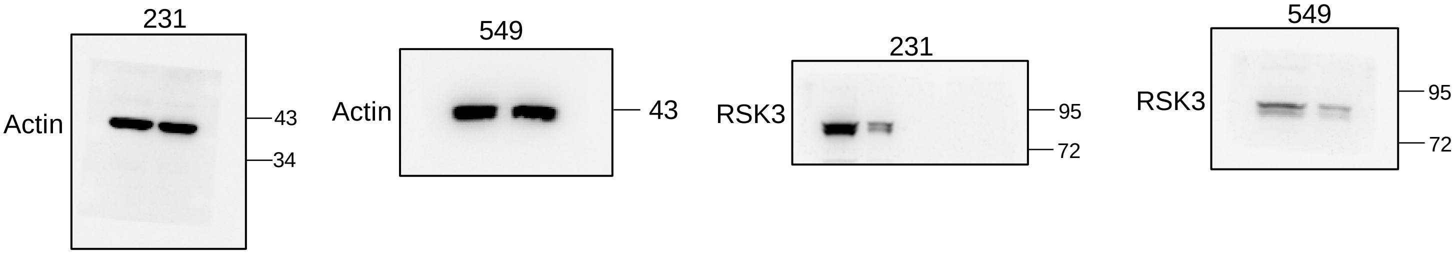

Supplement: Supplementary file 1 — Supplementary Information [file 41467_2019_14083_MOESM1_ESM.pdf]
